# *Irish Veterinary Journal *goes open access

**DOI:** 10.1186/2046-0481-64-1

**Published:** 2011-03-31

**Authors:** Michael L Doherty

**Affiliations:** 1 School of Agriculture, Food Science and Veterinary Medicine, University College Dublin, Belfield, Dublin 4, Ireland

## 

As Editor-in-Chief, I am delighted to announce the re-launch of *Irish Veterinary Journal *with BioMed Central, The Open Access Publisher, as part of their increasing veterinary portfolio. This is indeed an historical moment in the history of *Irish Veterinary Journal*, which was first published in November 1946. In this electronic age that we live in, it is interesting to note that the publication of the first issue was delayed by a post-war shortage of paper.

The first scientific paper published in the *Irish Veterinary Journal *was a study of blood serum parameters in cattle, horses, sheep and pigs in Ireland by W. Burke McAleer; a paper that emerged from his PhD thesis in the Veterinary College in Dublin. In its early years an editorial committee managed the journal and there was no mention of an individual Editor until the mid-1960s when J.K. Kealy was listed officially as Editor. B.T. Farrelly, who presided over the IVJ until 1980, soon followed him. Dr. Pat Hartigan became Editor in 1981 and worked tirelessly as peer-review Editor for many years until 2006. Management of publishing over this period was carried out by Irish Veterinary Publications Ltd. and more recently by IFP Media Ltd. for the new *Veterinary Ireland *organisation.

The move to Biomed Central heralds an exciting chapter in the history of the journal. *Irish Veterinary Journal*, with the support of *Veterinary Ireland *- the Irish professional representative organisation, is delighted to become part of the greater BMC publishing family and hopes to offer a new voice for high quality veterinary research internationally. Along with my Deputy Editors, Simon More, John Mee and Grace Mulcahy and expert editorial board, I would like to wish the new *Irish Veterinary Journal *every success.

*Guím gath rath ar Iris Tréidliachta Éireann nua*!

Michael Doherty

Editor-in-Chief

Irish Veterinary Journal

